# Sex-related differences in severe native valvular heart disease: the ESC-EORP Valvular Heart Disease II survey

**DOI:** 10.1093/eurheartj/ehae523

**Published:** 2024-08-30

**Authors:** Julia Mascherbauer, Andreas Kammerlander, Christian Nitsche, Jeroen Bax, Victoria Delgado, Arturo Evangelista, Cecile Laroche, Aldo Pietro Maggioni, Julien Magne, Alec Vahanian, Bernard Iung

**Affiliations:** Department of Internal Medicine 3, University Hospital St. Pölten, Dunantplatz 1, 3100 St. Poelten, Karl Landsteiner University of Health Sciences, Krems, Austria; Department of Internal Medicine 2, Division of Cardiology, Medical University of Vienna, Vienna, Austria; Department of Internal Medicine 2, Division of Cardiology, Medical University of Vienna, Vienna, Austria; Department of Cardiology, Leiden University Medical Center, Leiden, The Netherlands; Heart Institute, Germans Trias i Pujol University Hospital, Badalona, Spain; Department of Cardiology, Hospital Universitari Vall d'Hebron, Barcelona, Spain; EURObservational Research Programme, European Society of Cardiology, Sophia-Antipolis, France; ANMCO Research Center, Heart Care Foundation, Firenze, Italy; Department of Cardiology, Dupuytren University Hospital 2, Limoges, France; Université Paris-Cité, 75006 Paris, France; Bichat Hospital, APHP, and INSERM LVTS 1148, Université Paris-Cité, Paris, France

**Keywords:** Sex differences, Valvular heart disease

## Abstract

**Background and Aims:**

To assess sex differences in disease characteristics and treatment of patients with severe native valvular heart disease (VHD) included in the VHD II EURObservational Research Programme.

**Methods:**

A total of 5219 patients were enrolled in 208 European and North African centres and followed for 6 months [41.2% aortic stenosis (AS), 5.3% aortic regurgitation (AR), 4.5% mitral stenosis (MS), 21.3% mitral regurgitation (MR), 2.7% isolated right-sided VHD, 24.9% multiple left-sided VHD]. Indications for intervention were considered concordant if corresponding to class I recommendations specified in the 2012 ESC or 2014 AHA/ACC VHD guidelines.

**Results:**

Overall, women were older, more symptomatic, and presented with a higher EuroSCORE II. Bicuspid aortic valve and AR were more prevalent among men while mitral disease, concomitant tricuspid regurgitation (TR), and AS above age 65 were more prevalent among women. On multivariable regression analysis, concordance with recommended treatment was significantly poorer in women with MS and primary MR (both *P* < .001). Age, patient refusal, and decline of symptoms after conservative treatment were reported significantly more often as reasons to withhold the intervention in females. Concomitant tricuspid intervention was performed at a similar rate in both sexes although prevalence of significant TR was significantly higher in women. In-hospital and 6-month survival did not differ between sexes.

**Conclusions:**

(i) Valvular heart disease subtype varied between sexes; (ii) concordance with recommended intervention for MS and primary MR was significantly lower for women; and (iii) survival of men and women was similar at 6 months.

## Structured Graphical Abstract

**Structured Graphical Abstract:**
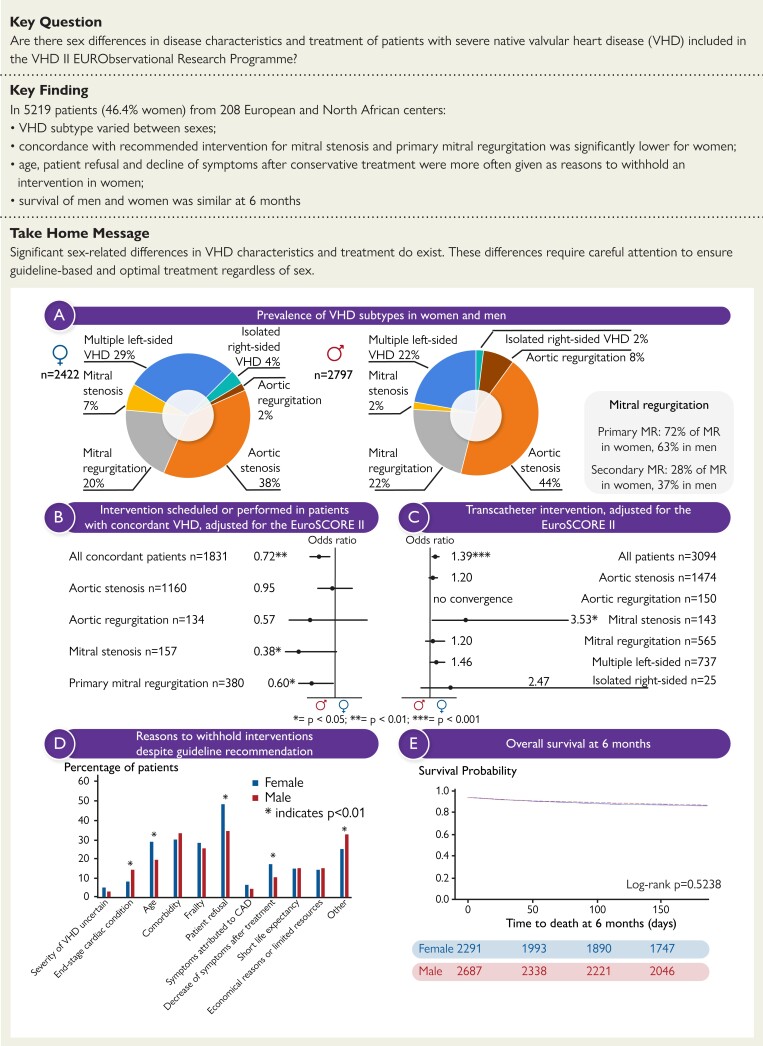
(*A*) Valvular heart disease (VHD) subtypes stratified by sex; (*B*) forest plots displaying women’s odds ratios for ‘Intervention scheduled or performed’ and (*C*) ‘Transcatheter intervention’ in patients with concordant VHD, adjusted for the EuroSCORE II; (*D*) reasons to withhold interventions despite guideline recommendation; (*E*) Kaplan–Meier curves for overall survival at 6 months, stratified by sex. CAD, coronary artery disease; MR, mitral regurgitation.

**See the editorial comment for this article ‘Unveiling sex differences in valvular disease care and outcomes', by P. Lancellotti *et al*., https://doi.org/10.1093/eurheartj/ehae524.**

## Introduction

Valvular heart disease (VHD) represents a major disease burden, affecting >2% of the European population and accounting for 0.4% of global deaths among elderly adults.^1–3^ Management according to guideline recommendations has improved over time, potentially driven by the introduction of novel, less invasive treatment options.^3,4^ Yet, recent data have highlighted important sex disparities, particularly regarding delayed presentation and treatment of women.^5,6^ However, sex differences in VHD are poorly studied. Most studies have focused on differences in outcomes following surgical or transcatheter therapies while more recent studies also evaluated epidemiology and pathophysiology of VHD, which may allow for a more focused approach to diagnosis and management.

In 2001, the ESC for the first time took a step to systemically collect data across European countries to assess VHD prevalence and management.^3^ Since then, several milestone developments dramatically changed the management options in VHD patients, which led to significant adaptions of the respective guideline recommendations.^7,8^

The present analyses were drawn from the VHD II survey designed by the EURObservational Research Programme (EORP) of the European Society of Cardiology (ESC), which was conducted in 2017.^4^ The VHD II survey aimed to analyse actual management of VHD in a large contemporary cohort of patients with native VHD and to assess concordance of European practice with guideline recommendations. The analyses presented in this manuscript assessed sex-specific differences regarding frequency, management, and outcomes of severe native VHD as well as underlying causative factors.

## Methods

### Study design

Details on the survey and data collection have been extensively presented elsewhere.^4^ In brief, 222 European and North African VHD centres (university, public, and private centres with or without onsite cardiac surgery or interventional cardiology) consecutively enrolled 7247 individuals with severe VHD or previous valvular intervention during a 3-month period in 2017. At 6 months, data on vital status, hospitalizations for cardiac reasons, and new valvular interventions were collected. The present study was restricted to the 5219 patients with severe native VHD. The primary endpoint was the final therapeutic decision for surgical or transcatheter intervention determined during the index hospitalization or outpatient visit. Drug prescription and indications for diagnostic/therapeutic procedures were left to the discretion of the attending physician. For patients undergoing intervention during the study period, the type of intervention performed and in-hospital mortality were collected. The survey was overseen by an Executive Committee and managed by the EORP department of the ESC, which was also responsible for study management, data quality control, and statistical analyses.

### Patients

The studied cohort consisted of patients with severe native VHD as defined by echocardiography using an integrative approach according to guidelines.^7,8^ Investigators were asked to include all consecutive hospitalized patients and/or a complete sample of outpatients presenting to the outpatient clinic 1 day each week (as selected by the centre). Patients were included in the present analysis if they fulfilled the following criteria: signed informed consent, age ≥ 18 years, severe native VHD. Patients with previous valvular interventions were excluded. Other exclusion criteria were acute infective endocarditis, enrolment in a valve intervention study affecting management, and VHD related to complex congenital heart disease.

### Classification of valvular heart disease

Single left-sided VHD was defined as severe VHD affecting a single valve without concomitant moderate or severe VHD on the other ipsilateral valve and subclassified as aortic stenosis (AS), aortic regurgitation (AR), mitral stenosis (MS), or mitral regurgitation (MR). The association of severe left-sided native VHD with a moderate or severe VHD lesion on the other ipsilateral valve (according to echocardiographic criteria) was classified as multiple left-sided VHD. Isolated right-sided VHD was defined as severe tricuspid or pulmonary VHD without any severe left-sided VHD. Patients presenting with native VHD who had undergone a previous valvular intervention on another valve were not taken into account.

### Statistical analysis

Descriptive analyses were performed. Continuous variables were reported as mean with standard deviation (SD) and categorical variables as percentages. Comparisons between groups were performed with a χ2 test or a Fisher’s exact test if any expected cell count was less than five for categorical variables and a Kruskal–Wallis test for continuous variables. Concordance with guidelines was analysed in patients with AS and a mean gradient > 40 mmHg, severe AR, MS with a valve area ≤ 1.5 cm2 or mean gradient > 5 mmHg, and severe primary MR and expressed by the percentage of patients in whom intervention was performed or scheduled among symptomatic patients, which corresponds to conditions fulfilling class I recommendations for intervention according to both 2012 ESC/European Association for Cardio-Thoracic Surgery (EACTS) and 2014 American Heart Association/American College of Cardiology (AHA/ACC) guidelines which were applicable during the survey period.^7,8^ We performed a logistic regression analysis of clinical management strategies adjusted for the EuroSCORE II which investigated sex-related differences regarding scheduled/performed interventions, the use of transcatheter approach, interventions scheduled, interventions withheld, and intervention scheduled or performed among concordant patients. In addition, interaction between sex and type of valve disease was tested. Survival of male and female patients was compared using Kaplan–Meier curves. Cox regression analyses using the endpoints cardiac death at 6 months and all-cause death were performed and adjusted for the EuroSCORE II and the region of the treating centre. These analyses were performed for the overall cohort and separately for patients who underwent interventions and those who were treated conservatively. Interaction between sex and type of valve disease was also tested. A two-sided *P*-value of <.05 was considered statistically significant. All analyses were performed with SAS statistical software version 9.4 (SAS Institute Inc., Cary, NC, USA).

## Results

### Patient characteristics

*Table 1* lists baseline characteristics stratified by sex. In total, 2797 (53.6%) male and 2422 (46.4%) female patients were enrolled, of whom 9 (0.4%) had an ongoing pregnancy during the survey. When compared to men, women were older (71.5 ± 14.3 vs. 69.0 ± 13.7 years), had fewer previous coronary interventions (11.0% vs. 17.8%), higher rates of atrial fibrillation (27.1% vs. 22.2%), were more symptomatic (NYHA functional classes III and IV, 42.2% and 5.5% vs. 32.7% and 5.1%), and presented with a higher EuroSCORE II (3.7 ± 5.8 vs. 3.2 ± 5.1). Overall, women showed more signs of age-associated impairment as compared to men (limited mobility: 9.0% vs. 5.8%, dementia: 2.1% vs. 1.0%, and Charlson’s index 4.1 ± 3.0 and 3.9 ± 2.5).

**Table 1:** Baseline characteristics

	All patients *n* = 5219 (100%)	Female *n* = 2422 (46.4%)	Male *n* = 2797 (53.6%)
Age (years) (mean ± SD)	70.2 (±14.0)	71.5 (±14.3)	69.0 (±13.7)
BMI (kg/m²) (mean ± SD)	27.5 (±5.0)	27.4 (±5.5)	27.5 (±4.5)
EuroSCORE II	3.4 (±5.4)	3.7 (±5.8)	3.2 (±5.1)
Inpatient hospitalization	3678/5219 (70.5%)	1722/2422 (71.1%)	1956/2797 (69.9%)
Previous coronary intervention	761/5199 (14.6%)	265/2411 (11.0%)	496/2788 (17.8%)
Percutaneous coronary intervention	619/756 (81.9%)	232/264 (87.9%)	387/492 (78.7%)
CABG	196/758 (25.9%)	49/265 (18.5%)	147/493 (29.8%)
Previous valve intervention	0/5214 (0.0%)	0/2420 (0.0%)	0/2794 (0.0%)
Active smoking	575/5219 (11.0%)	139/2422 (5.7%)	436/2797 (15.6%)
Hypertension	3627/5219 (69.5%)	1681/2422 (69.4%)	1946/2797 (69.6%)
Hyperlipidaemia	2511/5219 (48.1%)	1122/2422 (46.3%)	1389/2797 (49.7%)
Diabetes	1190/5218 (22.8%)	549/2422 (22.7%)	641/2796 (22.9%)
Non-insulin treated	877/1190 (73.7%)	381/549 (69.4%)	496/641 (77.4%)
Insulin treated	313/1190 (26.3%)	168/549 (30.6%)	145/641 (22.6%)
Creatinine clearance (mL/min) (mean ± SD)	71.0 (±32.5)	63.0 (±29.1)	78.0 (±33.8)
Chronic pulmonary disease	609/5178 (11.8%)	251/2404 (10.4%)	358/2774 (12.9%)
Liver dysfunction	120/5109 (2.3%)	40/2369 (1.7%)	80/2740 (2.9%)
Porcelain aorta	67/4885 (1.4%)	29/2264 (1.3%)	38/2621 (1.4%)
Chest deformation	36/5097 (0.7%)	24/2361 (1.0%)	12/2736 (0.4%)
Limited mobility	379/5219 (7.3%)	218/2422 (9.0%)	161/2797 (5.8%)
Previous myocardial infarction	524/5173 (10.1%)	193/2403 (8.0%)	331/2770 (11.9%)
Lower limb atherosclerosis	249/4755 (5.2%)	82/2196 (3.7%)	167/2559 (6.5%)
Cancer	454/5219 (8.7%)	208/2422 (8.6%)	246/2797 (8.8%)
Previous thoracic radiation	92/5219 (1.8%)	68/2422 (2.8%)	24/2797 (0.9%)
Dementia	79/5219 (1.5%)	50/2422 (2.1%)	29/2797 (1.0%)
Ongoing pregnancy		9/2422 (0.4%)	
Charlson’s index (mean ± SD)	4.0 (±2.7)	4.1 (±3.0)	3.9 (±2.5)
NYHA class			
I	964/5219 (18.5%)	339/2422 (14.0%)	625/2797 (22.3%)
II	2042/5219 (39.1%)	928/2422 (38.3%)	1114/2797 (39.8%)
III	1938/5219 (37.1%)	1022/2422 (42.2%)	916/2797 (32.7%)
IV	275/5219 (5.3%)	133/2422 (5.5%)	142/2797 (5.1%)
Congestive heart failure at the time of examination	1218/5219 (23.3%)	609/2422 (25.1%)	609/2797 (21.8%)
Angina pectoris (CCS)	859/5219 (16.5%)	380/2422 (15.7%)	479/2797 (17.1%)
Previous stroke/TIA	372/5219 (7.1%)	182/2422 (7.5%)	190/2797 (6.8%)
Pre-operative ECG rhythm			
Sinus	3702/5214 (71.0%)	1678/2420 (69.3%)	2024/2794 (72.4%)
Atrial fibrillation/flutter	1274/5214 (24.4%)	655/2420 (27.1%)	619/2794 (22.2%)
Paced	198/5214 (3.8%)	73/2420 (3.0%)	125/2794 (4.5%)
Other	40/5214 (0.8%)	14/2420 (0.6%)	26/2794 (0.9%)

BMI, body mass index; SD, standard deviation; CABG, coronary artery bypass graft; TIA, transient ischaemic attack.

### Valvular heart disease characteristics

Baseline echocardiographic data and characteristics of VHD are displayed in *Table 2*. Overall, men were more likely to present with a severely reduced left ventricular ejection fraction (<30%: 3.2% vs. 6.9%) and more dilated left ventricular cavities—the latter no longer significant if adjusted for body surface area.

**Table 2:** Baseline imaging data

	All patients *n* = 5219 (100%)	Female *n* = 2422 (46.4%)	Male *n* = 2797 (53.6%)
LV end-diastolic diameter (mm)	52.5 (±9.4)	49.3 (±8.3)	55.3 (±9.5)
LV end-diastolic diameter indexed to body surface area (mm/m^2^)	19.5 (±5.2)	19.1 (±5.1)	19.8 (±5.3)
LV end-systolic diameter (mm)	36.2 (±9.8)	33.2 (±8.5)	38.9 (±10.1)
LV end-systolic diameter indexed to body surface area (mm/m^2^)	28.2 (±5.2)	28.3 (±5.3)	28.1 (±5.2)
LV ejection fraction (%)			
0–19	50/5125 (1.0%)	12/2375 (0.5%)	38/2750 (1.4%)
20–29	217/5125 (4.2%)	65/2375 (2.7%)	152/2750 (5.5%)
30–39	398/5125 (7.8%)	143/2375 (6.0%)	255/2750 (9.3%)
40–49	668/5125 (13.0%)	258/2375 (10.9%)	410/2750 (14.9%)
50–59	1472/5125 (28.7%)	694/2375 (29.2%)	778/2750 (28.3%)
≥60	2320/5125 (45.3%)	1203/2375 (50.7%)	1117/2750 (40.6%)
Systolic pulmonary artery pressure (mmHg)			
<30	39/178 (21.9%)	14/82 (17.1%)	25/96 (26.0%)
30–55	84/178 (47.2%)	46/82 (56.1%)	38/96 (39.6%)
>55	55/178 (30.9%)	22/82 (26.8%)	33/96 (34.4%)
Native valve disease			
Degenerative	3448/5085 (67.8%)	1567/2358 (66.5%)	1881/2727 (69.0%)
Rheumatic	599/5085 (11.8%)	404/2358 (17.1%)	195/2727 (7.2%)
Previous endocarditis	32/5085 (0.6%)	10/2358 (0.4%)	22/2727 (0.8%)
Inflammatory	11/5085 (0.2%)	7/2358 (0.3%)	4/2727 (0.1%)
Congenital	309/5085 (6.1%)	90/2358 (3.8%)	219/2727 (8.0%)
Secondary mitral regurgitation	374/5085 (7.4%)	140/2358 (5.9%)	234/2727 (8.6%)
Other	312/5085 (6.1%)	140/2358 (5.9%)	172/2727 (6.3%)
Aortic valve disease	3760/5219 (72.0%)	1673/2422 (69.1%)	2087/2797 (74.6%)
Bicuspid valve disease	465/3069 (15.2%)	134/1351 (9.9%)	331/1718 (19.3%)
Aortic stenosis	3082/3760 (82.0%)	1412/1673 (84.4%)	1670/2087 (80.0%)
Severe aortic stenosis	2085/2987 (69.8%)	971/1374 (70.7%)	1114/1613 (69.1%)
Aortic regurgitation	678/3760 (18.0%)	261/1673 (15.6%)	417/2087 (20.0%)
Severe aortic regurgitation	365/678 (53.8%)	88/261 (33.7%)	277/417 (66.4%)
Mitral valve disease	2913/5219 (55.8%)	1509/2422 (62.3%)	1404/2797 (50.2%)
Mitral stenosis	509/2913 (17.5%)	373/1509 (24.7%)	136/1404 (9.7%)
Severe mitral stenosis	387/494 (78.3%)	288/364 (79.1%)	99/130 (76.2%)
Mitral regurgitation	2404/2913 (82.5%)	1136/1509 (75.3%)	1268/1404 (90.3%)
Severe mitral regurgitation	1268/2404 (52.7%)	579/1136 (51.0%)	689/1268 (54.3%)
Tricuspid regurgitation	1778/5219 (34.1%)	920/2422 (38.0%)	858/2797 (30.7%)
No/mild	667/1778 (37.5%)	307/920 (33.4%)	360/858 (42.0%)
Moderate	686/1778 (38.6%)	373/920 (40.5%)	313/858 (36.5%)
Severe	425/1778 (23.9%)	240/920 (26.1%)	185/858 (21.6%)

LV, left ventricular.

**Figure 1:**
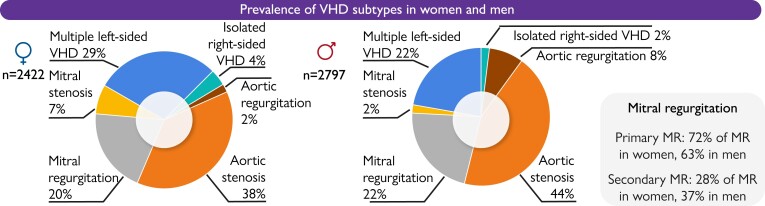
Valvular heart disease (VHD) subtypes stratified by sex

The presence of VHD subtypes stratified by sex is displayed in *Figure 1*. Of the 5219 patients with severe native VHD, degenerative valve disease was the most common aetiology in both men (69.0%) and women (66.5%). Overall, 3779 (72.4%) had single left-sided native VHD.^4^

Among patients with aortic valve (AV) disease (*n* = 3760), severe AS was found in 2085 (69.8% of AS); and severe AR was present in 365 patients (53.8% of AR cases). Men more often presented with severe AR and bicuspid AV. Overall, no significant difference between sexes regarding the proportion of severe AS was observed, while in patients older than 65, significant AS was more often documented in women than men.

Mitral valve disease was more frequently reported among women (62.3% vs. 50.2% in men). Among 2913 patients with mitral valve disease, severe MR was present in 1268 (52.7% of MR) with no difference in the prevalence between sexes.

Severe MS was documented in 387 (78.3% of MS) patients with similar distribution among sexes.

Of the 368 patients with secondary MR, 190 (51.6%) were classified as having ischaemic MR and 178 (48.4%) as having non-ischaemic MR.

Multiple left-sided VHD was present in 1297 patients (24.9% of native VHD), and isolated right-sided VHD was observed in 143 patients (2.7% of native VHD).

The presence of moderate or severe TR was significantly more common in women when compared to men (40.5% vs. 36.5% and 26.1% vs. 21.6%).

### Interventional treatment

A total of 1929 (37.0%) valve interventions were performed during the 3-month recruitment period, with a similar frequency in both sexes (36.8% [*n* = 892/2422] in women vs. 37.1% [*n* = 1037/2797] in men). After the 6-month follow-up, 47.9% (*n* = 2499/5219) had received a valve intervention, again, with a similar a frequency in both sexes (46.5% in women vs. 49.1% in men). During the course of the study, the following interventions were captured:

Aortic stenosis: A total of 554 female and 657 male patients with AS underwent intervention. Women were significantly older (75.6 ± 10.9 vs. 71.6 ± 12.4 years) and less often received concomitant procedures such as coronary artery bypass graft (CABG), aortic root replacement, ablation of atrial fibrillation, or left atrial appendage exclusion (15.5% vs. 24.7%). Men were more likely to receive a mechanical prosthesis as compared to women (24.4% vs. 16.2%). In contrast, a transcatheter approach was performed more often in women (42.4% vs. 33.3% in men). In-hospital mortality was 3.3% in women and 1.7% in men.
Aortic regurgitation: A total of 196 patients (69 female, 127 male; age 62.7 ± 16.0 years in females vs. 56.6 ± 15.6 years in males) underwent intervention for AR.
  A mechanical prosthesis was used in 34.6% of male and 21.7% of female patients, surgical repair or an autograft was used in 20.5% of men and 5.8% of women.
  Transcatheter treatment was rare and did not differ between sexes (5.8% in females vs. 1.6% in males). Regarding concomitant valve procedures women received mechanical mitral prostheses and stented mitral bioprostheses more frequently (21.7% vs. 7.1%; and 15.9% vs. 1.6%, respectively). Concomitant procedures such as CABG, aortic root replacement, ablation of atrial fibrillation, or left atrial appendage exclusion were more frequently performed in men (44.1% vs. 29.0%). In-hospital mortality was 1.6% in women and 0.9% in men.
Mitral stenosis: More women underwent intervention for MS than men (*n* = 160 vs. *n* = 55) without significant difference in age (56.8 ± 17.4 female vs. 59.2 ± 14.9 male). Among them, we observed a similar rate of mechanical prostheses (42.5% in women vs. 47.3% in men). Concomitant procedures such as CABG, ablation of atrial fibrillation, or left atrial appendage exclusion were more frequently performed in men (30.9% vs. 16.3%). In-hospital mortality was 2.1% in women vs. 0% in men.
Mitral regurgitation: In 339 women and 408 men with MR (age 71.4 ± 13.6 years in women vs. 68.3 ± 13.5 in men), an intervention was performed during the survey period. Surgical valve repair was more common in men (35.5% vs. 25.4%) with no significant difference in the use of mechanical prostheses (9.3% vs. 12.4%). The frequency of transcatheter treatment did not differ between sexes (8.0% in females vs. 8.6% in males). Concomitant procedures such as CABG, ablation of atrial fibrillation, or left atrial appendage exclusion were more frequently performed in men (35.0% vs. 22.7%). In-hospital mortality was 2.8% in women vs. 2.6% in men.
Isolated right-sided interventions: Isolated right-sided interventions were performed very rarely (8 women and 10 men). Three women and five men received surgical tricuspid valve repair, a tricuspid bioprosthetic valve was implanted in four women and four men. One woman underwent transcatheter tricuspid valve repair. One woman and one man received a pulmonic bioprosthesis.
Concomitant tricuspid intervention: Among all patients undergoing intervention for left-sided VHD, concomitant tricuspid intervention was performed at a similar frequency in both sexes (14.3% vs. 11.9% for women vs. men) although presence of moderate or severe TR was significantly more common in women (*Table 2*).

### Clinical management strategies

*Table 3* presents the logistic regression analysing sex-related differences in clinical management strategies among types of valve disease adjusted for the EuroSCORE II and the interaction between sex and type of valve disease. The interaction between sex and type of valve disease was not statistically significant across all analyses.

**Table 3:** Logistic regression analysis of clinical management strategies

	Adjusted for EuroSCORE II			
	Crude women OR [95% CI]	Adjusted women OR [95% CI]	*P*-value adjusted OR	Number of obs used
Intervention scheduled or performed				
All patients, *n* = 5216	0.82 [0.73;0.91]	0.82 [0.73;0.92]	.001	4737 (90.8%)
Aortic stenosis, *n* = 2149	0.83 [0.68;1.01]	0.88 [0.72;1.09]	NS	1948 (90.6%)
Aortic regurgitation, *n* = 279	0.73 [0.40;1.33]	0.69 [0.35;1.32]	NS	239 (85.7%)
Mitral stenosis, *n* = 234	0.75 [0.40;1.41]	0.62 [0.30;1.27]	NS	214 (91.5%)
Primary mitral regurgitation, *n* = 746	0.86 [0.64;1.15]	0.88 [0.64;1.20]	NS	673 (90.2%)
Secondary regurgitation, *n* = 368	1.04 [0.68;1.61]	0.97 [0.62;1.52]	NS	337 (91.6%)
Multiple left-sided, *n* = 1297	0.81 [0.65;1.01]	0.77 [0.61;0.98]	.030	1203 (92.8%)
Isolated right-sided, *n* = 143	0.39 [0.17;0.92]	0.32 [0.13;0.82]	.017	123 (86.0%)
Transcatheter approach				
All patients, *n* = 3327	1.50 [1.29;1.74]	1.39 [1.18;1.63]	<.001	3094 (93.0%)
Aortic stenosis, *n* = 1589	1.42 [1.16;1.74]	1.20 [0.95;1.50]	NS	1474 (92.8%)
Aortic regurgitation, *n* = 170	5.15 [0.69;38.15]	NA	NA	150 (88.2%)
Mitral stenosis, *n* = 150	3.86 [1.27;11.70]	3.53 [1.15;10.82]	.027	143 (95.3%)
Mitral regurgitation, *n* = 609	1.25 [0.79;1.96]	1.20 [0.72;2.01]	NS	565 (92.8%)
Multiple left-sided, *n* = 782	1.55 [1.14;2.12]	1.46 [1.05;2.03]	.025	737 (94.2%)
Isolated right-sided, *n* = 27	1.63 [0.26;10.10]	2.47 [0.37;16.48]	NS	25 (92.6%)
Intervention withheld				
All patients, *n* = 3287	1.41 [1.21;1.64]	1.45 [1.23;1.71]	<.001	2951 (89.8%)
Aortic stenosis, *n* = 1283	1.46 [1.12;1.90]	1.52 [1.14;2.02]	.004	1155 (90.0%)
Aortic regurgitation, *n* = 186	1.21 [0.42;3.53]	0.85 [0.21;3.40]	NS	156 (83.9%)
Mitral stenosis, *n* = 125	2.55 [1.07;6.08]	2.42 [0.91;6.42]	NS	108 (86.4%)
Mitral regurgitation, *n* = 747	0.93 [0.68;1.27]	0.98 [0.70;1.38]	NS	664 (88.9%)
Multiple left-sided, *n* = 827	1.26 [0.94;1.68]	1.31 [0.97;1.78]	NS	768 (92.9%)
Isolated right-sided, *n* = 119	1.25 [0.54;2.90]	1.08 [0.44;2.64]	NS	100 (84.0%)
Intervention scheduled or performed among concordant patients				
All patients, *n* = 2000	0.74 [0.60;0.91]	0.72 [0.58;0.90]	.004	1831 (91.6%)
Aortic stenosis, *n* = 1271	0.91 [0.69;1.19]	0.95 [0.70;1.28]	NS	1160 (91.3%)
Aortic regurgitation, *n* = 147	0.71 [0.28;1.78]	0.57 [0.21;1.55]	NS	134 (91.2%)
Mitral stenosis, *n* = 168	0.46 [0.20;1.08]	0.38 [0.15;1.00]	.049	157 (93.5%)
Primary mitral regurgitation, *n* = 414	0.64 [0.42;0.98]	0.60 [0.38;0.96]	.034	380 (91.8%)

CI, confidence interval; NA, not applicable; OR, odds ratio.

The overall rate of ‘intervention scheduled or performed’ was lower in women (odds ratio [OR], 0.82; 95% confidence interval [CI], 0.73; 0.92; *P* = .001). While no significant sex-related differences in scheduled or performed interventions were observed for AS, AR, MS, and MR, women were less often scheduled for or underwent interventions for multiple left-sided VHD (OR, 0.77; 95% CI 0.61; 0.98; *P* = .030) and isolated right-sided VHD (OR, 0.32; 95% CI 0.13; 0.82; *P* = .017) during the study period.

**Figure 2:**
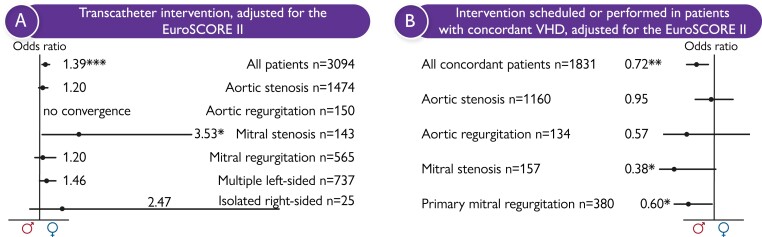
(*A*) ‘Transcatheter intervention' in patients with concordant VHD, adjusted for the EuroSCORE II. Forest plot displaying women's odds ratios for ‘Transcatheter intervention', adjusted for the EuroSCORE II. A transcatheter approach was significantly more often performed in women. While no significant difference between sexes was found with respect to TAVI use for the treatment of AS in the adjusted analysis, transcatheter interventions were more frequently documented for females with mitral stenosis and with multiple left-sided VHD. (*B*) Forest plot displaying women's odds ratios for ‘Intervention scheduled or performed' in patients with concordant VHD, adjusted for the EuroSCORE II. The overall rate of ‘Interventions scheduled or performed’ was significantly lower in women. Particularly in women with MS and primary MR, the analysis showed a significantly lower rate of interventional treatment in concordance with respective guidelines after adjustment for the EuroSCORE II

A transcatheter approach was more often performed in women than men (OR, 1.39; 95% CI 1.18; 1.63; *P* < .001). While no significant difference between sexes was found in transcatheter AV implantation (TAVI) use for the treatment of AS in the adjusted analysis (OR, 1.20; 95% CI 0.95; 1.50; *P* = .126), transcatheter interventions were more frequently documented for females with mitral stenosis (OR, 3.53; 95% CI 1.15; 10.82; *P* = .027) and with multiple left-sided VHD (OR, 1.46; 95% CI 1.05; 2.03; *P* = .025) (*Figure 2A*).

Regarding treatment in concordance with class I indications of the 2012 ESC/EACTS guidelines and the 2014 AHA/ACC guidelines,^7,8^ the overall rate of interventions scheduled or performed was documented to be significantly lower in female patients (OR, 0.72; 95% CI 0.58; 0.90; *P* = .004). Particularly in women with MS (OR, 0.38; 95% CI 0.15; 1.00; *P* = .049) and primary MR (OR, 0.60; 95% CI 0.38; 0.96, *P* = .034), the analysis showed a significantly lower rate of interventional treatment in concordance with respective guidelines after adjustment for the EuroSCORE II (*Figure 2B*).

### Decisions for interventions

*Table 4* details indications for interventions reported by the attending physician during the index hospitalization or outpatient visit. Significant sex-related differences for the reason for no intervention were reported.

**Table 4:** Reasons for therapeutic decisions

	All patients *n* = 5219 (100%)	Female *n* = 2422 (46.4%)	Male *n* = 2797 (53.6%)	*P*-value
Reason to withhold intervention				
Severity of VHD uncertain	34/805 (4.2%)	22/424 (5.2%)	12/381 (3.1%)	.151
End-stage cardiac condition	90/806 (11.2%)	35/424 (8.3%)	55/382 (14.4%)	.006
Age	197/806 (24.4%)	122/424 (28.8%)	75/382 (19.6%)	.003
Comorbidity	254/806 (31.5%)	127/424 (30.0%)	127/382 (33.2%)	.315
Frailty	217/806 (26.9%)	120/424 (28.3%)	97/382 (25.4%)	.352
Patient refusal	335/806 (41.6%)	204/424 (48.1%)	131/382 (34.3%)	<.001
Symptoms attributed to CAD	45/806 (5.6%)	28/424 (6.6%)	17/382 (4.5%)	.184
Decrease of symptoms after treatment	114/806 (14.1%)	73/424 (17.2%)	41/382 (10.7%)	.008
Short life expectancy	121/806 (15.0%)	63/424 (14.9%)	58/382 (15.2%)	.897
Economical reasons or limited resources	120/806 (14.9%)	61/424 (14.4%)	59/382 (15.4%)	.673
Other	231/806 (28.7%)	107/424 (25.2%)	124/382 (32.5%)	.024
Transcatheter approach	967/3327 (29.1%)	500/1484 (33.7%)	467/1843 (25.3%)	<.001
Reason for transcatheter approach				
Age	619/967 (64.0%)	335/500 (67.0%)	284/467 (60.8%)	.045
Contraindication for surgery	93/967 (9.6%)	39/500 (7.8%)	54/467 (11.6%)	.047
High risk for surgery	546/967 (56.5%)	284/500 (56.8%)	262/467 (56.1%)	.827
Intermediate risk for surgery	180/967 (18.6%)	95/500 (19.0%)	85/467 (18.2%)	.750
Patient preference	329/967 (34.0%)	186/500 (37.2%)	143/467 (30.6%)	.031

CAD, coronary artery disease; VHD, valvular heart disease.

**Figure 3:**
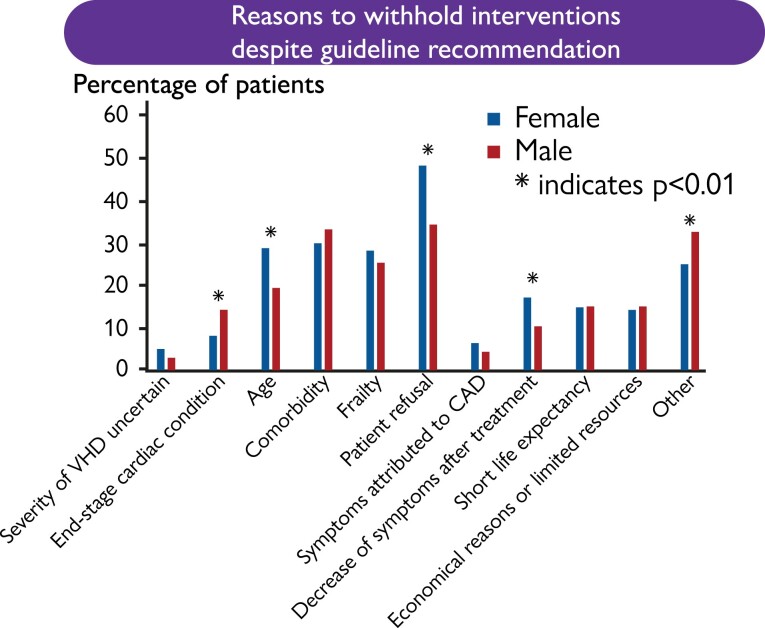
Reasons to withhold interventions despite guideline recommendation. Investigators reported age, patient refusal, and decline of symptoms after conservative treatment more often as reasons to withhold the intervention in women as compared to men (*P* < .01 for all). In men, however, end-stage cardiac condition and other reasons were more often reported as the reason to withhold invasive treatment (*P* < .01)

Among patients who had an indication for intervention but were withheld, investigators reported age, patient refusal, and decline of symptoms after conservative treatment more often as reasons to withhold the intervention in women as compared to men (*P* < .01 for all). In men, however, end-stage cardiac condition was more often reported as the reason to withhold invasive treatment (*P* = .006) (*Figure 3*).

Overall, transcatheter interventions were more frequently used in women than men when an intervention was scheduled or performed (33.7% [*n* = 500/1484] vs. 25.3% [*n* = 467/1843], *P* < .001). In women, age (67.0% vs. 60.8%, *P* = .045) and patient preference (37.2% vs. 30.6%, *P* = .031) were more often given as reasons for a transcatheter based therapy when compared to men. Conversely, contraindication to surgery was more often reported as reason for a transcatheter approach in men as compared to women (11.6% vs. 7.8%, *P* = .047).

### Survival

**Figure 4:**
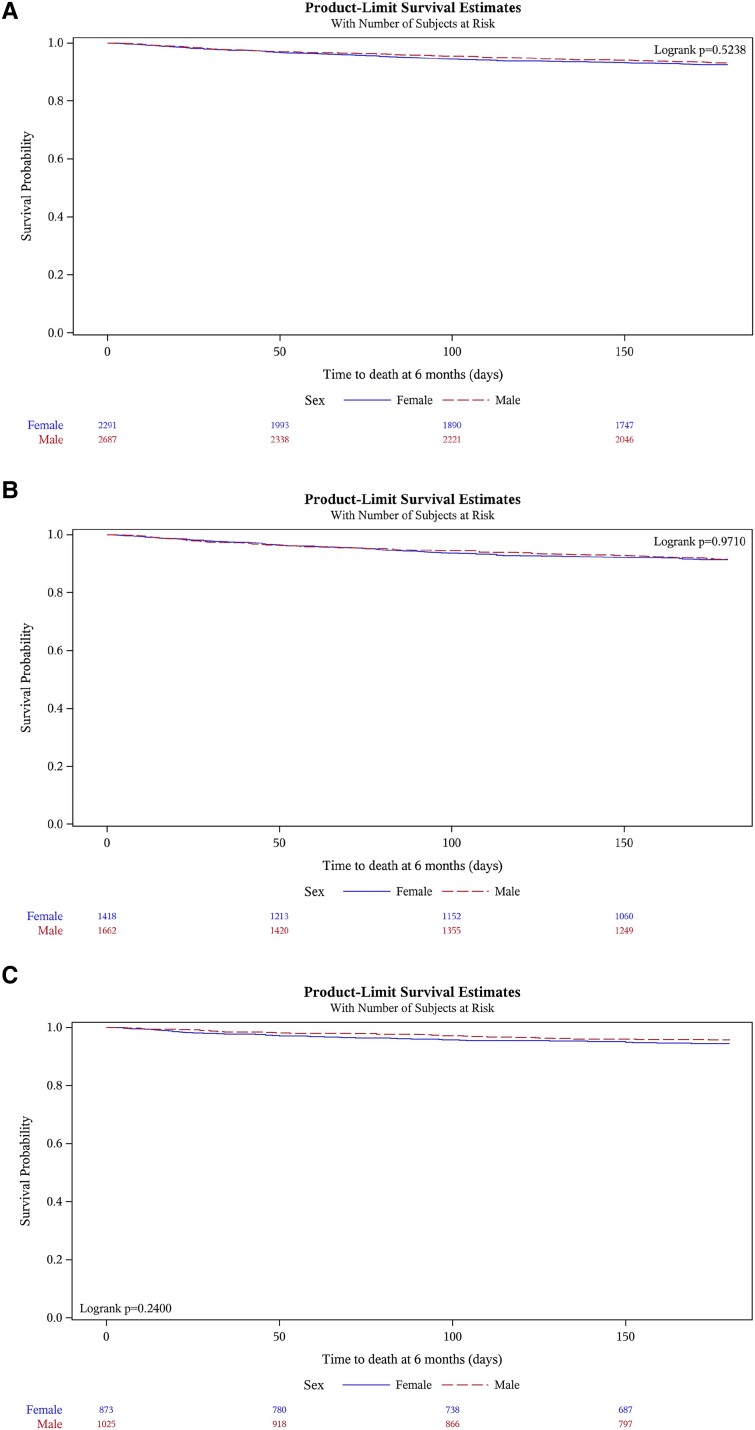
(*A*) Kaplan–Meier estimates displaying overall survival differences between men and women. (*B*) Kaplan–Meier estimates displaying overall survival differences between men and women who were treated conservatively. (*C*) Kaplan–Meier estimates displaying overall survival differences between men and women who underwent intervention

In-hospital survival was excellent and did not differ between sexes as demonstrated in the Kaplan–Meier curves (97.5% women and 98.0% men, *P* = .302, *Figure 4A*). During the 6-month follow-up, 6.8% (*n* = 308/4523) of participants deceased without significant differences between sexes (6.9% [*n* = 143/2074] in women vs. 6.7% [*n* = 165/2449] men, *P* = .834). Similarly, no survival differences were observed between men and women who underwent interventions (*Figure 4B*) and those who were treated conservatively (*P* = .971) (*Figure 4C*).

To assess survival in more depth, Cox regression analyses on all-cause death and cardiovascular death at 6 months were carried out and adjusted for sex, region of treatment, EuroSCORE II, and interaction between sex and type of valve disease. These analyses were performed for all patients (*Table 5*), patients who underwent interventions (*Table 6*), and those who were treated conservatively (*Table 7*). Across the broad spectrum of VHD subtypes—and within the entire cohort—no significant association of sex and mortality was identified (*Structured Graphical Abstract*).

**Table 5:** Cox regression analysis for the endpoints all-cause death and cardiac death at 6 months, adjusted for EuroSCORE II and region of treatment

	Adjusted for region		Adjusted for region and EuroSCORE II		
	Women HR [95% CI]	No. of obs used	Women HR [95% CI]	*P*-value	No. of obs used
All-cause death					
All patients, *n* = 5219	1.05 [0.86;1.28]	4978 (95.4%)	1.03 [0.84;1.28]	.761	4519 (86.6%)
Aortic stenosis, *n* = 2152	1.17 [0.85;1.62]	2062 (95.8%)	1.12 [0.79;1.59]		1865 (86.7%)
Aortic regurgitation, *n* = 279	1.59 [0.31;8.06]	272 (97.5%)	2.54 [0.43;15.00]		232 (83.2%)
Mitral stenosis, *n* = 234	0.64 [0.12;3.46]	201 (85.9%)	NC		183 (78.2%)
Mitral regurgitation, *n* = 1114	0.82 [0.53;1.27]	1056 (94.8%)	0.82 [0.52;1.31]		959 (86.1%)
Multiple left-sided, *n* = 1297	0.95 [0.67;1.35]	1251 (96.5%)	0.96 [0.67;1.38]		1163 (89.7%)
Isolated right-sided, *n* = 143	1.13 [0.37;3.46]	136 (95.1%)	0.95 [0.29;3.10]		117 (81.8%)
Cardiac death at 6 months					
All patients, *n* = 5219	1.07 [0.80;1.43]	4980 (95.4%)	1.04 [0.77;1.41]	.796	4519 (86.6%)
Aortic stenosis, *n* = 2152	1.18 [0.76;1.83]	2062 (95.8%)	1.15 [0.72;1.85]		1864 (86.6%)
Aortic regurgitation, *n* = 279	3.58 [0.50;25.46]	272 (97.5%)	6.99 [0.63;78.05]		232 (83.2%)
Mitral stenosis, *n* = 234	NC	203 (86.8%)	NC		185 (79.1%)
Mitral regurgitation, *n* = 1114	1.02 [0.54;1.93]	1056 (94.8%)	0.93 [0.48;1.79]		959 (86.1%)
Multiple left-sided, *n* = 1297	0.75 [0.44;1.26]	1252 (96.5%)	0.75 [0.43;1.30]		1162 (89.6%)
Isolated right-sided, *n* = 143	1.81 [0.19;17.08]	135 (94.4%)	2.10 [0.22;19.86]		117 (81.8%)

HR, hazard ratio; CI, confidence interval; obs, objects; NC, not calculated.

**Table 6:** Cox regression analysis on patients who underwent interventions for the endpoints all-cause death and cardiac death at 6 months

	Adjusted for region		Adjusted for region and EuroSCORE II		
	Women HR [95% CI]	No. of obs read	Women HR [95% CI]	*P*-value	No. of obs read
All-cause death					
All patients, *n* = 1929	1.11 [0.74;1.66]	1898 (98.4%)	1.10 [0.72;1.68]	.673	1756 (91.0%)
Aortic stenosis, *n* = 866	1.55 [0.84;2.85]	851 (98.3%)	1.54 [0.79;3.01]		778 (89.8%)
Aortic regurgitation, *n* = 93	2.31 [0.20;26.03]	93 (100.0%)	20.31 [0.14;2973.33]		83 (89.2%)
Mitral stenosis, *n* = 109	NC	97 (89.0%)	NC		94 (86.2%)
Mitral regurgitation, *n* = 367	0.66 [0.28;1.55]	365 (99.5%)	0.57 [0.23;1.40]		344 (93.7%)
Multiple left-sided, *n* = 470	1.14 [0.52;2.52]	468 (99.6%)	1.21 [0.54;2.74]		434 (92.3%)
Isolated right-sided, *n* = 24	NC	24 (100.0%)	NC		23 (95.8%)
Cardiac death at 6 months					
All patients, *n* = 1929	1.18 [0.51;2.73]	1898 (98.4%)	1.25 [0.50;3.11]	.636	1755 (91.0%)
Aortic stenosis, *n* = 866	2.59 [0.46;14.53]	851 (98.3%)	5.36 [0.57;50.11]		778 (89.8%)
Aortic regurgitation, *n* = 93	2.99 [0.18;48.63]	93 (100.0%)	NC		83 (89.2%)
Mitral stenosis, *n* = 109	NC	97 (89.0%)	NC		94 (86.2%)
Mitral regurgitation, *n* = 367	0.44 [0.09;2.20]	364 (99.2%)	0.19 [0.02;1.66]		343 (93.5%)
Multiple left-sided, *n* = 470	1.83 [0.33;10.02]	469 (99.8%)	2.45 [0.43;14.08]		434 (92.3%)
Isolated right-sided, *n* = 24	NC	24 (100.0%)	NC		23 (95.8%)

Hazard ratios are adjusted for region and EuroSCORE II.

HR, hazard ratio; obs, objects; CI, confidence interval; NC, not calculated.

**Table 7:** Cox regression analysis of patients treated conservatively for the endpoints all-cause death and cardiac death at 6 months

	Adjusted for region		Adjusted for region and EuroSCORE II		
	Women HR [95% CI]	No. of obs read	Women HR [95% CI]	*P*-value	Number of obs read
All-cause death					
All patients, *n* = 3290	1.05 [0.84;1.33]	3080 (93.6%)	1.04 [0.81;1.33]	.757	2763 (84.0%)
Aortic stenosis, *n* = 1286	1.13 [0.77;1.66]	1211 (94.2%)	1.07 [0.70;1.62]		1087 (84.5%)
Aortic regurgitation, *n* = 186	1.16 [0.13;10.61]	179 (96.2%)	0.63 [0.02;17.54]		149 (80.1%)
Mitral stenosis, *n* = 125	0.58 [0.10;3.24]	104 (83.2%)	NC		89 (71.2%)
Mitral regurgitation, *n* = 747	0.89 [0.54;1.49]	691 (92.5%)	0.89 [0.51;1.54]		615 (82.3%)
Multiple left-sided, *n* = 827	0.86 [0.58;1.27]	783 (94.7%)	0.86 [0.57;1.30]		729 (88.1%)
Isolated right-sided, *n* = 119	1.38 [0.42;4.56]	112 (94.1%)	1.09 [0.30;3.90]		94 (79.0%)
Cardiac death at 6 months					
All patients, *n* = 3290	1.08 [0.79;1.47]	3082 (93.7%)	1.06 [0.77;1.46]	.731	2764 (84.0%)
Aortic stenosis, *n* = 1286	1.17 [0.74;1.86]	1211 (94.2%)	1.14 [0.69;1.87]		1086 (84.4%)
Aortic regurgitation, *n* = 186	3.67 [0.23;58.69]	179 (96.2%)	1.46 [0.04;57.35]		149 (80.1%)
Mitral stenosis, *n* = 125	NC	106 (84.8%)	NC		91 (72.8%)
Mitral regurgitation, *n* = 747	1.22 [0.59;2.50]	692 (92.6%)	1.15 [0.56;2.35]		616 (82.5%)
Multiple left-sided, *n* = 827	0.62 [0.36;1.09]	783 (94.7%)	0.62 [0.34;1.11]		728 (88.0%)
Isolated right-sided, *n* = 119	1.90 [0.19;18.75]	111 (93.3%)	2.43 [0.24;25.02]		94 (79.0%)

Hazard ratios are adjusted for region and EuroSCORE II.

HR, hazard ratio; CI, confidence interval; obs, objects; NC, not calculated.

## Discussion

Valvular heart diseases are common, treatable, result in significant morbidity and mortality, and require a substantial allocation of health resources. The Global Burden of Disease Study 2017^2^ has previously estimated an enormous disease burden among older adults posed by calcific AV and degenerative mitral valve disease, particularly in high-income countries. However, sex-specific differences in the presentation, treatment, and outcome of VHD have so far been addressed in a limited number of studies.^6,9–14^

The VHD II data collection was undertaken in 2017. It prospectively enrolled 7247 patients with significant VHD across 28 European and North African countries and forms the basis of the present analysis.^4^ The survey was specifically designed to assess current clinical practice and guideline implementation regarding the management of patients with VHD.

### Sex differences in aortic valve disease

Aortic valve disease is common in high-income countries, with a more than two-fold greater incidence of AS than AR.^15^ Congenital bicuspid AV has been reported to be present in up to 2% of the population at birth, with a 3:1 male to female ratio.^16^ The present data support these previous findings by showing a two-fold higher proportion of bicuspid aortic disease and severe AR in men (*Table 2*). Previous data also reported a higher prevalence of AS among men.^17^ However, in the present survey, the overall distribution of severe AS was not different between sexes while in patients older than 65, significant AS was significantly more often documented in women than men.

Baseline characteristics and long-term outcomes of AS were previously shown to differ between sexes. Tribouilloy *et al*.^6^ recently published 5-year outcomes of a retrospective analysis of 2429 patients with severe AS (49.5% women), finding that at initial presentation women were older, with less comorbidities but more symptoms and higher pulmonary pressures than men. Similar observations were made in the present VHD II cohort. Women were significantly older, more symptomatic, presented with a higher EuroSCORE II, and showed more signs of age-associated impairment. Tribouilloy *et al*. also showed that after matching for age, women with severe AS had a lower 5-year survival than men despite their longer life expectancy in the general population. Similar findings have been reported by Bienjonetti-Boudreau *et al*. who studied 3632 patients (42% women) with at least mild AS. After adjustment for important confounders (age, diabetes, hypertension, renal and coronary disease, chronic pulmonary disease, symptoms, AV area, mean gradient, and indexed stroke volume), female sex was associated with greater mortality, possibly related to a greater likelihood of AV interventions in men.^9^ In comparison with these studies, the present survey is limited by its follow-up period of only 6 months, during which no significant sex-related differences in survival were observed.

Female patients with severe AS have previously been reported to receive surgical AV replacement (AVR) less frequently, and, if it was performed, women suffered more symptoms and were treated at more severe stages of disease than men.^11^ In the present survey, such findings were not confirmed. After adjustment for the EuroSCORE II, a multivariable logistic regression analysis of treatment strategies showed that women with concordant indications for treatment were as likely as men to be scheduled for or undergo intervention for AS (*Table 3*). However, treating physicians more often documented that treatment was withheld in women with AS. As reasons for withholding treatment age, patient refusal, and decrease of symptoms after conservative treatment were significantly more often documented for women (*Figure 3*).

Following TAVI, women have been shown to experience better survival rates than men.^18,19^ In particular, a lower rate of patient-prosthesis mismatch after TAVI might contribute to this finding. More recent analyses of the PARTNER II S3 study^20^ and the CENTER collaboration^21^ showed no apparent sex-specific differences in survival or stroke on multivariable analyses, possibly reflecting the changing demography of patients, use of newer-generation valves and delivery systems, and better valve sizing techniques. However, a survival benefit of surgical AVR or TAVI in AS may not extend to all patient populations. In the TOPAS study, women had similar outcomes as men in the medically managed subset but markedly higher mortality in the subset of patients undergoing AV intervention.^12^ This sex-specific disparity might relate to presence or absence of concomitant coronary artery disease and differential myocardial damage due to pressure overload. Unfortunately, the present survey lacks granular data on low-flow low-gradient AS. The use of TAVI and overall survival over 6 months were not different between sexes for AS.

### Sex differences in mitral valve disease

Mitral valve disease is frequent and associated with significant morbidity and mortality.^22^ The most common cause of chronic primary MR is mitral valve prolapse, with a trend towards higher prevalence among women.^23^ Secondary MR is caused either by left ventricular remodelling on the basis of ischaemic or non-ischaemic aetiologies or left atrial and annular remodelling.^22^ Men have been documented to suffer an almost two-fold higher risk than women for heart failure with reduced ejection fraction.^24^ In the present patient population, however, severe MR was found at similar rates in men and women.

With respect to MS, female sex is a predictor of incident mitral annulus calcification.^25^ In addition, rheumatic heart disease has been reported to be much more prevalent among women.^26^ In the present cohort, mitral valve disease was more frequently documented among women, however, with no sex difference in the presence of severe stenosis or severe regurgitation.

Data on the impact of sex on outcome in patients undergoing surgical or interventional treatment of mitral valve disease are scarce and conflicting.^13,27,28^ At the time of mitral intervention, women were reported to be older, with more comorbidities, and higher symptomatology than men.^29,30^ However, after propensity matching, outcomes between men and women were not significantly different suggesting that the sex differences in unmatched studies are related to late presentation and comorbidities. Notably, women comprised <50% of the interventional cohort in most studies, and <30% of patients treated with minimally-invasive techniques.^31^

In the present study, the female proportion among patients who underwent surgery for MS was three times higher than for men, without significant differences in age. Similar rates of mechanical prostheses were reported, and no differences in operative mortality.

Women suffering MR in the present survey were older and less likely to receive surgical valve repair while the use of mechanical prostheses or transcatheter treatment did not differ between sexes. In-hospital mortality was similar. Class I indications to intervene on women with primary MR were significantly less often followed in the present cohort. This may, to some extent, be explained by the fact that in MR treatment indications are based on the presence of left ventricular enlargement.^32,33^ Guideline-recommended cut-off values indicating the necessity of treatment are not systematically indexed to body size, resulting in less women to meet criteria for intervention.^23,32,34^ In a retrospective analysis of more than 8000 individuals with mitral valve prolapse, left heart dimensions were larger in females after normalization to body surface area, highlighting the need for sex-specific or indexed left ventricular and atrial diameter cut-offs.^14^ Although we cannot draw definitive conclusions from the present VHD II data as to the reasons why class I indications to intervene on women with primary MR were less often followed in the present cohort, the interpretation of ‘still normally sized’ heart cavities may have contributed. Furthermore, old age of women with primary severe MR, which did not qualify them for surgical intervention, anatomical conditions that made them poor candidates for procedures and the lack of transcatheter edge-to-edge repair opportunity may have contributed to the observed differences. Similarly to MR, women with concordant indications for the treatment of MS were also less likely to undergo intervention. This finding, although speculative, may be related to the clinical condition of the respective female patients that did not qualify them for surgical interventions, and, again, the lack of transcatheter treatment opportunities.

### Sex differences in tricuspid valve disease

Historical data reported a strong association with female sex and prevalence of significant TR such that by the eighth decade women with TR outnumbered men by 4:1.^35^ Two more recent studies confirmed a higher prevalence of significant functional TR among females, which was associated with older age and atrial dilatation. Atrial fibrillation was reported to be associated with TR in females but not in males. After propensity score matching, no significant sex-related difference in mortality in patients with significant TR was found.^36,37^

In the present cohort, moderate or severe TR was significantly more common in women when compared to men, however, the difference between sexes was much smaller than previously reported (40.5% vs. 36.5% for moderate TR; 26.1% vs. 21.6% for severe TR; *P* across groups < .001). Among all patients undergoing intervention for left-sided VHD, concomitant tricuspid intervention was performed at a similar frequency in both sexes although presence of moderate or severe TR was significantly more common in women.

### Limitations

The VHD II survey is not a comprehensive population-based epidemiological study but a voluntary survey. Representativeness is therefore suboptimal, and selection bias cannot be excluded. Nationwide registries based on hospital discharge codes ensure representativeness but do not provide information on either the severity of VHD or the symptomatic status of the patients and cannot be used to assess the application of guidelines. Thus, the inclusion of 208 centres from 28 European and North African countries in the VHD II survey results in data from a wide spectrum of healthcare structures to provide an in-depth insight into the contemporary presentation and management of VHD. A large variety of healthcare systems and types of medical centres were part of the survey. Among them, there may have been significant variety in resource availability and treatment possibilities, which may have impacted reported results. However, the present analysis was not intended to focus on geographical/regional differences but give an overview on sex-specific differences in the treatment and outcome of severe native VHD across all Europe. It has furthermore to be noted that women tend to have a longer life expectancy than men. Thus, it remains uncertain whether our finding of a similar survival at 6 months may reflect an actual survival disadvantage of women. Unfortunately, we were unable to perform an observed-to-expected mortality assessment since patients were recruited by a large number of centres from many different countries. In addition, the 2017 survey may not be reflective of current practice given the more recent randomized controlled trials of transcatheter device therapies that have since been reported. The use of transcatheter therapies is likely to be significantly higher now—and presumably—will continue to increase.

In the absence of onsite data monitoring, there was no direct control on consecutive patient inclusion and data accuracy. This was partly compensated for by multiple checks for consistency with the case report form and queries sent to investigators when data were missing or inconsistent. It is not possible to exclude survival bias in the analysis of 6-month follow-up, which was not available in 10.9% of patients.

## Conclusions

The present analysis provides sex-specific details in VHD across all Europe, which can be summarized as follows: (i) VHD subtype varied between sexes; (ii) concordance with recommended intervention for MS and primary MR was significantly lower for women; (iii) age, patient refusal, and decline of symptoms after conservative treatment were more often given as reasons to withhold an intervention in women; (iv) concomitant tricuspid intervention was performed at a similar rate in both sexes although prevalence of significant TR was significantly higher in women; and (v) survival of men and women was similar at 6 months.

## Data Availability

The VHD II survey was an international prospective, multicentre, observational study. All the National Cardiac Society members of the ESC were invited to participate. Participating centres were accepted on a voluntary basis through appointment by national coordinators. The data, analytical methods, and study materials will be available on reasonable request.

## Pre-registered Clinical Trial Number

None supplied.

## Appendix

### Group author list—all names from all sub-groups should be downstreamed as collaborators to PubMed): EORP VHD II Registry Investigators Group

**Includes all Principal Investigators of the EORP VHD II Registry Investigators Group:** Albania: Durres: Eliverta Zera, Tirana: Ervina Shirka, Tirana: Elona Dado, Austria: Vienna: Julia Mascherbauer, Vienna: Maria Heger, Azerbaijan: Baku: Fuad Samadov, Baku: Yasmin Rustamova, Belgium: Aalst: Gery Van Camp, Brussels: Agnès Pasquet, Gent: Nico Van de Veire, Jette: Bernard Cosyns, Czech Republic: Stepanka Sindelarova, Plzen: Katerina Linhartova, Denmark: Copenhagen: Nikolaj Ihlemann, Egypt: Assiut: Hosam Hasan Ali, Aswan: Mohamed Hassan, Beni Suef: Yasser Ahmed Abdelhady Ahmed, Cairo: Yasser Sadek, Cairo: Magdy Abdelhamid, Fayoum: Haytham Soliman Ghareeb, Giza: Ghada Kazamel, Finland: Helsinki: Minna Margareta Kylmälä, Kuopio: Anu Turpeinen, Turku: Antti Saraste, France: Amiens: Christophe Tribouilloy, Angers: Loic Biere, Cholet: Martin Audonnet, Helfaut: Hélène Bardet, Limoges: Julien Magne, Lomme: Sylvestre Marechaux, Marseille: Gilbert Habib, Marseille: Franck Thuny, Paris: Jean-Luc Monin, Paris: Bernard Iung, Rennes: Erwan Donal, Rennes: Renaud Gervais, Rouen: Fabrice Bauer, Saint-Denis: David Attias, Kulumbegov Saint-Brieuc: Magalie Daudin, Tours: Anne Bernard, FYR Macedonia: Skopje: Elizabeta Srbinovska Kostovska, Skopje: Marija Gjerakaroska Radovikj Germany: Bad Oeynhausen: Werner Scholtz, Bonn: Georg Nickenig, Essen: Tienush Rassaf, Greifswald: Kristin Lehnert, Gütersloh: Fikret Er, Jena: Paul-Christian Schulze, Mannheim: Ibrahim Akin, Regensburg: Lars S. Maier, Rostock: Huseyin Ince, Wuppertal: Marc Vorpahl, Zwickau: Holger Sigusch, Great Britain: Uxbridge: Shelley L. Rahman Haley, Greece: Athens: Ioannis Kanakakis, Athens: Andreas Katsaros, Athens: Konstantinos Spargias, Athens: Konstantinos Lampropoulos, Ioannina: Vasileios Bellos, Ioannina: Michail Papafaklis, Patras: Athanasios Makris, Thessaloniki: Vasileios Kamperidis, Thessaloniki: Vlasis Ninios, Thessaloniki: Vasileios Sachpekidis, Voula: Leonidas Poulimenos, Hungary: Budapest: Tünde Borsányi, Budapest: Zoltan Jarai, Budapest: András Zsáry, Kosovo: Pristina: Gani Bajraktari, Latvia: Liepaja: Iveta Sime, Riga: Andrejs Erglis, Ventspils: Gita Rancane, Lithuania: Kaunas: Vaida Mizariene, Vilnius: Greta Radauskaitė, Malta: Msida: Daniela Cassar Demarco, Netherlands: Leiden: Victoria Delgado, Poland: Katowice: Katarzyna Mizia-Stec, Warsaw: Piotr Szymanski, Portugal: Almada: Isabel Joao, Braga: Nuno Antunes, Faro: Dina Bento, Lisbon: Goncalo Cardoso, Lisbon: Ana Galrinho, Porto: Carla Sousa, Porto: Sofia Cabral, Vila Real: Sofia Silva Carvalho, Viseu: Ines Almeida, Romania: Baia Mare: Calin Pop, Brasov: Diana Tint, Bucharest: Bogdan A. Popescu, Bucharest: Andreea Popescu, Galati: Lucica Grigorica, Iasi: Catalina Arsenescu-Georgescu, Pitesti: Catalin Usurelu, Timisoara: Adina Ionac, Iasi: Antoniu Octavian Petris, Russian Federation: Astrakhan: Dmitry Kozmin, Khabarovsk: Oleg Kulumbegov, Khabarovsk: Krasnodar: Natalia Spiropulos, Krasnodar: Sergey Boldyrev, Moscow: Alex Zotov, Saint Petersburg: Olga Irtyuga, Samara: Dmitrii V. Kuznetsov, Serbia: Belgrade: Natasa Markovic Nikolic, Sremska Kamenica: Anastazija Stojsic-Milosavljevic, Valjevo: Dusan Ruzicic, Slovakia: Bratislava: Iveta Simkova, Kosice: Gabriel Valocik, Presov: Martin Studencan, Spain: Barcelona: Arturo Evangelista, Barcelona: Sergio Moral, Benalmadena: María José Molina Mora, El Palmar: Gonzalo de la Morena Valenzuela, Las Palmas de Gran Canaria: Irene Menduiña Gallego, Madrid: Cristina Mitroi, Madrid: Teresa Alberca Vela, Madrid: Ariana González-Gómez, San Javier: Luis Caballero Jimenez, Santa Cruz de Tenerife: Noelia Castro, Sevilla: Irene Méndez Santos, Sevilla: David Villagomez Villegas, Valencia: Rafael Payá, Valencia: Esther Esteban, Vigo: Cristina Iglesia-Carreno, Vigo: Francisco Calvo Iglesias, Turkey: Karesi-Balikesir: Abdullah Orhan Demirtas, Edirne: Gokay Taylan, Eregli: Naile Eris Gudul, Eskisehir: Kadir Ugur Mert, Eskisehir: Gurbet Ozge Mert, Istanbul: Sait Mesut Dogan, Istanbul: Şükrü Arslan, Istanbul: Nurten Sayar, Karesi-Balikesir: Tarik Yildirim, Sanliurfa: Asuman Yesilay, Trabzon: Muhammet Rasit Sayin, Zonguldak: Turgut Karabag.
